# Protective Efficacy of Rhomboid-Like Protein 3 as a Candidate Antigen Against *Eimeria maxima* in Chickens

**DOI:** 10.3389/fmicb.2021.614229

**Published:** 2021-05-05

**Authors:** Chen Chen, Di Tian, Junzhi Su, Xiaoqian Liu, Muhammad Ali A. Shah, Xiangrui Li, Lixin Xu, Ruofeng Yan, Xiaokai Song

**Affiliations:** ^1^MOE Joint International Research Laboratory of Animal Health and Food Safety, College of Veterinary Medicine, Nanjing Agricultural University, Nanjing, China; ^2^Nanjing Ringpai Vet Hospital Co., Ltd., Nanjing, China; ^3^Department of Pathobiology, Pir Mehr Ali Shah Arid Agriculture University, Rawalpindi, Pakistan

**Keywords:** coccidiosis, *Eimeria maxima*, rhomboid-like protein, protective efficacy, candidate antigen

## Abstract

Avian coccidiosis brings tremendous economic loss to the poultry industry worldwide. The third generation vaccine, including subunit and DNA vaccines, exhibited promising developmental prospects. In a previous study, we found rhomboid-like protein 3 of *Eimeria maxima* (EmROM3) was involved in infections by *Eimeria* species. However, the protective efficacy of EmROM3 against *Eimeria maxima* (*E. maxima*) remains unknown. In this study, chickens were intramuscularly immunized with the recombinant protein EmROM3 (rEmROM3) or pVAX1-EmROM3 to determine the EmROM3-induced immune response. The induced humoral immune response was determined by measuring serum IgG antibody levels in immunized chickens. The induced cellular immune response was detected by measuring the transcription level of immune related cytokines and the proportion of T cell subsets of the immunized chickens. Finally, the protective efficacy of the EmROM3 vaccine against *E. maxima* was evaluated by immunization-challenge trials. Results revealed that the purified rEmROM3 reacted with chicken anti-*E. maxima* serum. The recombinant plasmid of pVAX1-EmROM3 was transcribed and translated in the injected muscle from the vaccinated chickens. In experimental groups, the IgG titers, proportions of CD4^+^ and CD8^+^ T cells, and transcription level of splenic cytokines were significantly increased compared with the control groups. The immunization-challenge trial revealed that immunization with rEmROM3 or pVAX1-EmROM3 led to restored weight gain, alleviated enteric lesion, decreased oocyst output as well as the higher anticoccidial index (ACI), indicating partial protection against *E. maxima*. These results indicate that EmROM3 is an effective candidate antigen for developing novel vaccines against infection by *E. maxima*.

## Introduction

Avian coccidiosis is an enteric disease caused by the *Eimeria* species and affects the worldwide poultry industry. It results in malabsorption, poor weight gain, and impaired feed conversion (Chapman and Jeffers, 2014; Sun et al., 2014). Infection of chicken coccidia contributes to an annual economic loss of more than $3 billion USD, including production losses and the costs of prevention and treatment (Blake and Tomley, 2014). At present, methods for controlling this disease mainly depend on in-feed chemoprophylaxis. However, the application of anticoccidial drugs is strictly restricted due to public concerns about the impacts of drug residues on food safety, the continual emergence of drug resistance, and the expensive costs related to developing new drugs (Pastor-Fernández et al., 2018). Vaccination with live virulent or attenuated strains leaves no chemical residues and seems a feasible control strategy, but owing to the inherent limitations of large-scale manufacturing and high costs, as well as potential pathogenicity, make live parasite vaccination an improvable control method. Other alternative strategies have been suggested as ways of preventing damage from chicken coccidia (Song et al., 2015a). More recently, third generation vaccines using gene derived pathogens as antigens have been the subject of a great deal of interest as they avoid the disadvantages of chemoprophylaxis and live vaccines (Wu et al., 2004; Konjufca et al., 2008; Song et al., 2010, 2015b).

Rhomboid-like proteins (ROMs) are a family of intramembrane serine proteases with diverse biological functions in different species (Dowse et al., 2005; Lastun et al., 2016; Shilo, 2016). In Apicomplexans, ROMs activity is involved in cleaving transmembrane adhesins from the surface of the zoites, thus resulting in a complete entry into host cells during parasite invasion (Dowse and Soldati, 2005; Urban, 2006; Rugarabamu et al., 2015). In *Toxoplasma gondii*, TgROM2 was reported to cleave to the chimeric proteins of TgMIC2 and TgMIC12 (Dowse et al., 2005). In *Plasmodium*, PfROMs 1 and 4 were found to be able to cleave to most of the reported adhesins proteins involved in host invasion by *Plasmodium* (Baker et al., 2006). In *Eimeria tenella*, ROM3 was reported to be involved in the cleavage of EtMIC4 (microneme protein 4) (Zheng et al., 2014). Therefore, ROMs are thought to be of critical importance for host cell invasion. Another study tested ROMs as antigen candidates and reported that they exhibited promising protective efficacy (Wang et al., 2009; Song et al., 2010).

We have previously found that EmROM3 might be involved in the cleavage of the microneme protein (unpublished data); however, the protective efficacy of EmROM3 against *Eimeria maxima* remains unknown. In the present study, we detected the cell-mediated immune responses and humoral immune responses activated by recombinant protein and the eukaryotic expression plasmid of EmROM3 in chickens. Then the protective efficacy of EmROM3 was evaluated in forms of recombinant protein (rEmROM3) and eukaryotic expression plasmid (pVAX1-EmROM3) in challenge trials. These observations suggest that EmROM3 could be a vaccine candidate antigen for controlling *E. maxima* infection.

## Materials and Methods

### Animals, Parasite, Plasmid, and Antiserum

Hy-Line layer chickens that were 1-day old were raised in strictly sterilized cages. Feed and water without anticoccidial drugs were provided *ad libitum*. SD rats were purchased from Qinglongshan animal breeding farm, Nanjing, China. *E. maxima* was stored at 4°C and propagated every 2 months. Prokaryotic expression vector pET-32a and eukaryotic expression vector pVAX1 were purchased from Novagen (Darmstadt, Germany) and Invitrogen (Carlsbad, CA, United States), respectively. All animal studies and protocols were approved by the Committee on Experimental Animal Welfare and Ethics of Nanjing Agricultural University.

Chicken antiserum against *E. maxima* was prepared following the previous report (Song et al., 2017). In brief, 14-day-old chickens were orally infected with 1 × 10^4^ of *E. maxima* at an interval of 7 days with five doses in total. Blood samples were collected for antiserum titer determination by indirect enzyme linked immunosorbent assay (ELISA) at the fifth dose (Liu et al., 2018). After the titer was beyond 2^6^, *E. maxima* antiserum was collected by heart blood collection and stored at −70°C for further use. Negative control serum was collected from chickens with no infection.

### Cloning and Expression Plasmid Construction of EmROM3

Extraction of *E. maxima* total RNA was conducted utilizing the Total RNA Kit I (OMEGA). Reverse transcription (RT) reaction was performed to produce cDNA with primers designed based on the sequence in GenBank (KR815509). The primers were designed with the software “Primer Premier 5” (Premier, Canada) with restriction enzyme-anchored (underlined): *Eco*RI anchored forward primer, 5′-CGGAATTCATGTCCGATATCGAGT-3′; *Xho*I anchored reverse primer, 5′-CCCTCGAGTTATGCGCACCCCATGGGC-3′. A PCR assay was carried out with the following procedure: an initial denaturation at 94°C for 5 min, followed by 35 cycles at 94°C for 30 s, 55°C for 30 s, and 72°C for 45 s. The PCR products were cloned into prokaryotic expression vector of pET-32a and then transformed into *Escherichia coli* BL21. Endonuclease digestion and sequence analysis were conducted to verify the recombinant product pET-32a-EmROM3. The complete open reading frame (ORF) of the target gene was aligned in GenBank databases with a basic alignment search tool (BLAST)^1^.

To construct the eukaryotic expression plasmid of pVAX1-EmROM3, EmROM3 was cleaved from pET-32a-EmROM3 by *Eco*RI and *Xho*I digestion. Subsequently, the EmROM3 fragment was ligated into pVAX1 digested with *Eco*RI and *Xho*I, producing pVAX1-EmROM3. Endonuclease digestion and sequencing were carried out to verify the recombinant products.

### Expression of EmROM3 Recombinant Protein and Production of Its Antiserum

Expression of rEmROM3 was carried out in host bacteria of *E. coli* BL21 and detected by SDS-PAGE. Subsequently, purification of rEmROM3 was conducted using Ni^2+^-nitrilotriacetic acid (Ni-NTA) column (GE Healthcare, United States). Endotoxin removal was performed using ToxinEraser^TM^ Endotoxin Removal Kit (GenScript, China) following the manufacturer’s instructions.

To produce antiserum against rEmROM3, SD rats were immunized by subcutaneous injection in the back with 250 μg rEmROM3 with adjuvant of FAC (Sigma). Fourteen days after the primary immunization, a booster immunization was given with adjuvant of FAI (Sigma), following one dose per week and a week apart for 4 weeks. After the fifth immunization, blood was collected from the fundus venous plexus of the rats. Subsequently, antiserum titer was determined by indirect ELISA and stored at −70°C. Meanwhile, pET-32a tag protein antiserum and negative serum were collected from the rat vaccinated pET-32a tag protein and naïve rat separately.

### Western Blot Analysis of EmROM3 Recombinant Protein With Chicken Antiserum Against *E. maxima*

SDS-PAGE was performed to transfer the purified recombinant protein to the nitrocellulose membrane. After blocking with BSA overnight at 4°C, the membranes were incubated with chicken antiserum against *E. maxima* (dilutions 1:100) at room temperature (RT) for 4 h. Meanwhile, a negative control was incubated with serum from uninfected chicken. After three times washes with PBST (Tris-buffer saline with 0.5% Tween-20), the membranes were incubated with horseradish peroxidase (HRP)-conjugated goat anti-chick IgG (dilutions 1:4500) at RT for 1.5 h. DAB (3,3′-diaminobenzidine tetrahydrochloride) kit (Boster Biotechnology) was used to detect the bound antibody.

### Detection of the Transcription and Expression of Recombinant Plasmid *in vivo* by RT-PCR and Western Bolt

Chickens (14 days old) were randomly divided into two groups (five chickens per group). One group was vaccinated with 100 μg of pVAX1-EmROM3 by leg intramuscular injection and the other one was injected with 100 μg of pVAX1 at the same site as empty vector control. Seven days later, all the chickens were slaughtered. Muscle samples were collected from the injection site and non-injection site for RT-PCR and Western bolt detection.

Total RNA was extracted from the muscle derived from the injected muscles to detect the transcription of the pVAX1-EmROM3 recombinant plasmid. The residual plasmid was removed by digestion with DNase I (TaKaRa). Using the produced RNA as a template, RT-PCR was carried out with the specific primers of the EmROM3 gene to detect the transcription of recombinant plasmid pVAX1-EmROM3. Meanwhile, the non-injected muscles were treated with the same method.

To detect the expression of the recombinant plasmid, after grinding in mortars, the injected muscles were treated with RIPA solution for 2 h. The supernatant was collected by centrifuging for 10 min (13,000 r/min). Western blot was carried out with rat anti-rEmROM3 serum as primary antibody to detect the expressed EmROM3. The serum of the unvaccinated rat was used as a negative control.

### Detection of the Immune Response Induced by EmROM3 in Chickens

#### Animal Immunization and Sample Collection

To detect the immune response induced by rEmROM3 or pVAX1-EmROM3, chickens (14 days old) were randomly assigned into six groups (30 chickens/group). An intramuscular injection was, respectively, performed with rEmROM3 (200 μg), pET-32a tag protein (200 μg), pVAX1-EmROM3 (100 μg), pVAX1 empty vector (100 μg), and PBS (200 μL, two groups) in the leg. A booster dose was administered in the same with as the first vaccination and at 21 days of age.

Seven days after the first vaccination and the booster vaccination, five birds from each group were killed by cervical-dislocation slaughter. The spleens were collected to determine CD4^+^ and CD8^+^ T cell subsets and mRNA level of cytokine separately. Meanwhile, blood samples were collected from the surplus 20 birds per group for specific antibody determination.

#### Determination of CD4^+^ and CD8^+^ T Cell Subsets by Flow Cytometric Assay

A single splenocyte was prepared, as described (Choi et al., 1999). Spleen cell suspensions (1 × 10^7^ and −1 × 10^8^ cell/mL) were collected into three centrifuge tubes (100 μL/10^7^ cells of each tube). One of them was dually stained by the addition of 2 μL anti-chicken CD3 mouse antibody (Southern Biotechnology Associates) and the same volume of anti-chicken CD4 mouse antibody (Southern Biotechnology Associates). Another tube was conducted using a dual dye with the same amount as the first tube of anti-chicken CD3 mouse antibody (Southern Biotechnology Associates) and anti-chicken CD8 mouse antibody (Southern Biotechnology Associates). Splenocytes in the last tube were divided into three equal parts with one no staining and the other two stained with 2 μL CD3 and CD4 or CD8. All tubes were incubated for 45 min at 4°C without light. Cell population was performed by a FACSCalibur flow cytometer (BD Biosciences, Franklin Lakes, NJ, United States).

#### Determination of Cytokines mRNA Level by qPCR

The transcription of IFN-γ, IL-2, IL-4, IL-17, TNFSF15, TGF-β, and IL-10 genes from the vaccinated chickens was measured using qPCR assay with internal control of GAPDH (Tian et al., 2017). In brief, splenocyte total RNA was extracted to generate the template cDNA for qPCR. A qPCR was carried out with the primers in Table 1. The qPCR reaction mixture contained 2 μL template cDNA, 0.4 μL primer, 10 μL 2 × ChamQ SYBR qPCR Master Mix, and 7.6 μL deionized water. PCR-grade water was used as a negative control in all qPCR assays. Each reaction was measured in triplicate. In this study, the 2^–ΔΔCT^ method was used to determine the relative quantification of cytokine gene mRNA compared with the internal control (n-fold change to the water control group) (Livak and Schmittgen, 2001). The amplification efficiencies of the cytokine genes and the internal control gene were measured by validation experiment with a series of diluted cDNA (Song et al., 2010).

**TABLE 1:** Primers used in qPCR.

RNA target	Primer sequence	Accession No.	Amplification efficiency (%)	Correlation coefficients (*r*^2^)
GAPDH	GGTGGTGCTAAGCGTGTTAT ACCTCTGTCATCTCTCCACA	K01458	100.74	0.9917
IL-2	TAACTGGGACACTGCCATGA GATGATAGAGATGCTCCATAAGCTG	AF000631	102.44	0.9921
IL-4	ACCCAGGGCATCCAGAAG CAGTGCCGGCAAGAAGTT	AJ621735	99.09	0.9936
IL-10	GGAGCTGAGGGTGAAGTTTGA GAAGCGCAGCATCTCTGACA	AJ621614	99.19	0.9923
IL-17	ACCTTCCCATGTGCAGAAAT GAGAACTGCCTTGCCTAACA	EF570583	100.24	0.9940
IFN-γ	AGCTGACGGTGGACCTATTATT GGCTTTGCGCTGGATTC	Y07922	103.07	0.9868
TGF-β	CGGGACGGATGAGAAGAAC CGGCCCACGTAGTAAATGAT	M31160	102.79	0.9815
TNFSF15	GCTTGGCCTTTACCAAGAAC GGAAAGTGACCTGAGCATAGA	NM_001024578	100.57	0.9930

#### Determination of Serum IgG Antibody Level by Indirect ELISA

Collection of the blood samples was conducted by cardiac puncture from each group, 7 days after the first and second immunization separately. Serum was collected for determining IgG antibody levels using indirect ELISA (Lillehoj et al., 2005). In brief, 150 μL rEmROM3 (10 μg/ml) was coated in flat-bottomed 96-well plates with 0.05 M carbonate buffer per well overnight at 5°C. After five times washes with PBS-T (PBS with 0.05% Tween-20), the plates were blocked with 200 μL 4.5% SMP (skim milk powder) for 5 h at RT. After another three washes with PBS-T, 100 μL serum samples diluted 1:50 in PBS-T were added to the plate to incubate with SMP at RT for 3 h. Serum from PBS-vaccinated chickens and unvaccinated chickens were set as blank control and negative control separately. The plate was incubated with 100 μL/well of HRP-conjugated goat anti chicken IgG antibody (dilutions 1:4500) at RT for 2.5 h after five times washes. Color development was conducted with 100 μL 3,3′,5,5′-tetramethylbenzidine (TMB) per well in dark at RT for 8 min and observed by a spectrophotometric method at OD_450_.

### Evaluation of Protective Efficacy Induced by EmROM3 Against *Eimeria maxima* in Chickens

Trial 1 and trial 2 were conducted to evaluate the protective efficacy of rEmROM3 and pVAX1-EmROM3, respectively. At 14 days old, chickens were randomly assigned into eight groups with 30 birds per group (Table 2). All birds were weighed and tagged individually. Two experimental groups were vaccinated intramuscularly with pVAX1-EmROM3 (100 μg) or rEmROM3 (200 μg) in the leg, separately. Six control groups were assigned as follows, below.

**TABLE 2:** Protective efficacy of recombinant EmROM3 vaccines against *E. maxima* challenge.

Trial	Groups	Weight gain (g)	Mean lesion score	Mean OPG × 10^5^	Oocyst decrease rate%	ACI
1	Unchallenged control	56.91 ± 10.24^a^	0 ± 0^a^	0 ± 0^a^	100^a^	200
	Challenged control	27.21 ± 8.52^c^	2.84 ± 0.88^c^	2.25 ± 0.94^c^	0^c^	79.41
	pET-32a tag protein control	29.46 ± 11.25^c^	2.66 ± 0.93^c^	2.15 ± 0.97^c^	4.44^c^	85.17
	rEmROM3	46.31 ± 8.07^b^	1.36 ± 0.76^b^	0.54 ± 0.50^b^	76.00^b^	166.77
2	Unchallenged control	79.32 ± 9.59^a^	0 ± 0^a^	0 ± 0^a^	100^a^	200
	Challenged control	39.28 ± 9.72^c^	2.83 ± 0.72^c^	2.81 ± 0.13^c^	0^c^	81.23
	pVAX1 control	38.19 ± 15.39^c^	2.75 ± 0.62^c^	2.80 ± 0.16^c^	0.36^c^	80.65
	pVAX1-EmROM3	60.00 ± 11.56^b^	1.32 ± 0.79^b^	0.61 ± 0.34^b^	78.29^b^	161.44

**N* = 30, the data = mean ± standard deviation. Trial 1 and trial 2 were conducted separately, the significance of data in these trials were incomparable. OPG was measured at 6 days after infection with *E. maxima*. Significant difference (*p* < 0.05) between data was annotated with different letters. No significant difference (*p* > 0.05) between data was annotated with the same letter.*

The challenged controls (two groups) and unchallenged controls (two groups) were injected with 200 μL of PBS separately. The vector tag protein control group and empty vector control group were injected with pET-32a tag protein (200 μg) and pVAX1 (100 μg), respectively. All the control groups were injected in the same way as the experimental groups. A second dose was given at 21 days of age. At 28 days old, except for the unchallenged control group, birds in other groups were challenged with 1 × 10^5^ *E. maxima* sporulated oocysts orally. Six days after the challenge, the chickens were weighed individually and killed for average weight gain calculation, lesion score determination, oocyst shedding counting, and anticoccidial index (ACI) calculation.

The efficacy of the vaccination was evaluated based on survival rate, oocyst shedding, enteric lesion score, weight gain, and ACI. The survival rate was determined by the amount of surviving chickens divided by the amount of initial chickens. Blind assessment on enteric lesion score was performed by an observer following the previous method (Johnson and Reid, 1970). Body weight gain was calculated by the terminal body weight subtracting the body weight when challenged. The oocyst counting per group was conducted by cage (total eight cages), following a method previously reported by Hodgson (1970). The percentage decrease in oocyst shedding was calculated as follows: (average oocysts amount of the challenged control group – that of the vaccinated groups)/oocysts amount of control group × 100%. ACI was calculated by the following formula: (relative rate of weight gain + survival rate) - (lesion index + oocyst index) (McManus et al., 1968).

### Statistical Analysis

Statistical analysis was conducted with the one-way ANOVA Duncan test in the SPSS statistical software (SPSS 20, SPSS Inc., Chicago, IL, United States). Differences between groups at *p* < 0.05 were considered significant.

## Results

### Cloning and Expression of EmROM3 Gene

**FIGURE 1:**
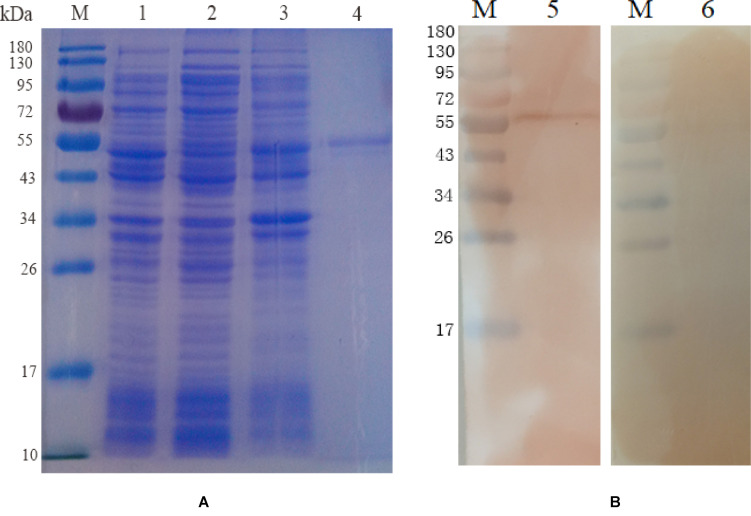
Expression and Western blot analysis of recombinant protein pET-32a EmROM3. **(A)** Expression and purification of recombinant protein pET-32a EmROM3. M: protein mid-molecular weight marker. Lane 1: Expression detection of empty vector pET-32a from the cell lysate. Lane 2: Expression detection of pET-32a-EmROM3 from the supernatant of the cell lysate. Lane 3: Expression detection of pET-32a-EmROM3 from inclusion bodies of the cell lysate. Lane 4: purified recombinant protein of pET-32a-EmROM3. **(B)** Western blot analysis of recombinant protein pET-32a EmROM3. M: protein mid-molecular weight marker. Lane 5: The purified recombinant protein of pET-32a-EmROM3 was recognized by the chicken serum against *E. maxima*. Lane 6: Serum from the non-infected chicken was used as a negative control.

The EmROM3 gene was cloned from *E. maxima* and ligated with eukaryotic expression vector pVAX1 and prokaryotic expression vector pET-32a separately, producing pET-32a-EmROM3 and pVAX1-EmROM3. Sequencing analysis revealed that ORF of EmROM3 is composed of 774 nucleotides, sharing nucleotide homology of 100% with the nucleotide sequence in GenBank (Sequence ID: KR815509). *E. coli* BL21 was used to express rEmROM3, following a purification through Ni-NTA column. The results of the SDS-PAGE assay revealed a single band of 46.3 kDa, which equals the totality of molecular weight of the EmROM3 protein and pET-32a tag protein (Figure 1A). Western blot showed that rEmROM3 was identified by anti*-E. maxima* chicken serum (Figure 1B).

### Transcription and Expression of pVAX1-EmROM3 in Chickens

**FIGURE 2:**
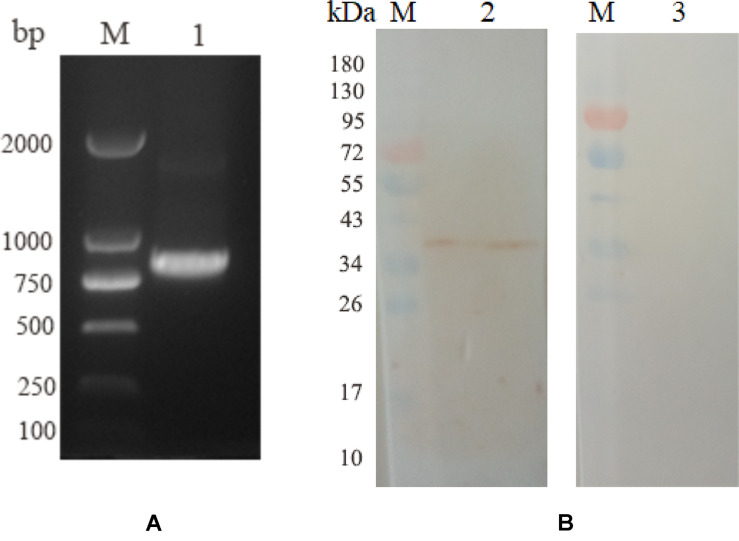
Transcription and expression of pVAX1-EmROM3 in chickens. **(A)** Transcription detection of pVAX1-EmROM3 in chickens by RT-PCR. M: DNA marker DL2000. Lane 1: EmROM3 was amplified from the pVAX1-EmROM3 injected chickens. **(B)** Expression detection of pVAX1-EmROM3 by Western blot assay. M: protein mid-molecular weight marker. Lane 2: pVAX1-EmROM3 injected muscle recognized by the anti-rEmROM3 rat serum. Lane 3: Serum from non-injected rat was used as a negative control.

Five birds were intramuscularly injected with pVAX1-EmROM3 to detect the Transcription and expression of pVAX1-EmROM3 in chickens. The other five chickens injected pVAX1 served as vector control. The representative results are shown in Figure 2A band of 774 bp was amplified from the injected muscle by RT-PCR, which equals the size of EmROM3 (Figure 2A). Western blot assay revealed that the rat antiserum against rEmROM3 recognized a band of approximately 33 kDa from the injected muscle (Figure 2B, Lane 2), which was slightly larger than the predicted molecular weight of EmROM3. While the negative rat serum did not recognize any band from the injected muscle (Lane 3). This observation indicated that pVAX1-EmROM3 was successfully transcribed and translated into the injected muscle.

### The Antibody Response Induced by rEmROM3 and Recombinant Plasmid of pVAX1-EmROM3 in Chickens

**FIGURE 3:**
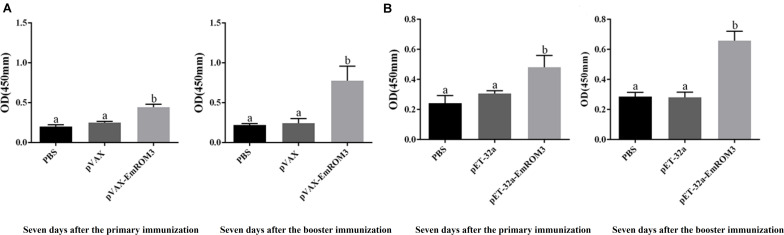
Specific IgG titers activated by recombinant protein pET-32a EmROM3 and recombinant plasmid pVAX1-EmROM3 in chickens. *N* = 20, the error bars = standard deviation. **(A)** Serum IgG titers induced by recombinant plasmid pVAX1-EmROM3. Left, induced serum IgG titers 7 days after the first immunization; Right, induced serum IgG titers 7 days after the booster immunization. **(B)** Serum IgG titers induced by recombinant protein pET-32a EmROM3. Left, induced serum IgG titers 7 days after the first immunization; Right, induced serum IgG titers 7 days after the booster immunization. Significant difference (*P* < 0.05) between groups is marked with different letters. No significant difference (*P* > 0.05) between groups is marked with the same letter.

Sera from the immunized chickens were measured by indirect ELISA for the determination of antibody titers. As depicted in Figure 3A, 7 days after the first and second vaccination, the IgG titer of the rEmROM3-immunized group was significantly higher compared to those of the PBS and pET-32a tag protein control group (*p* < 0.05). Similarly, immunization significantly prompted the serum IgG titer of the immunized group compared to the PBS control and pVAX1 vector control (*p* < 0.05) (Figure 3B). The titers between the PBS control group and pET-32a tag protein control group, as well as PBS control group and pVAX1 vector control group did not show a significant difference (*p* > 0.05).

### The Cellular Response Induced by the Recombinant Protein of pET-32a-EmROM3 and Recombinant Plasmid of pVAX1-EmROM3 in Chickens

**FIGURE 4:**
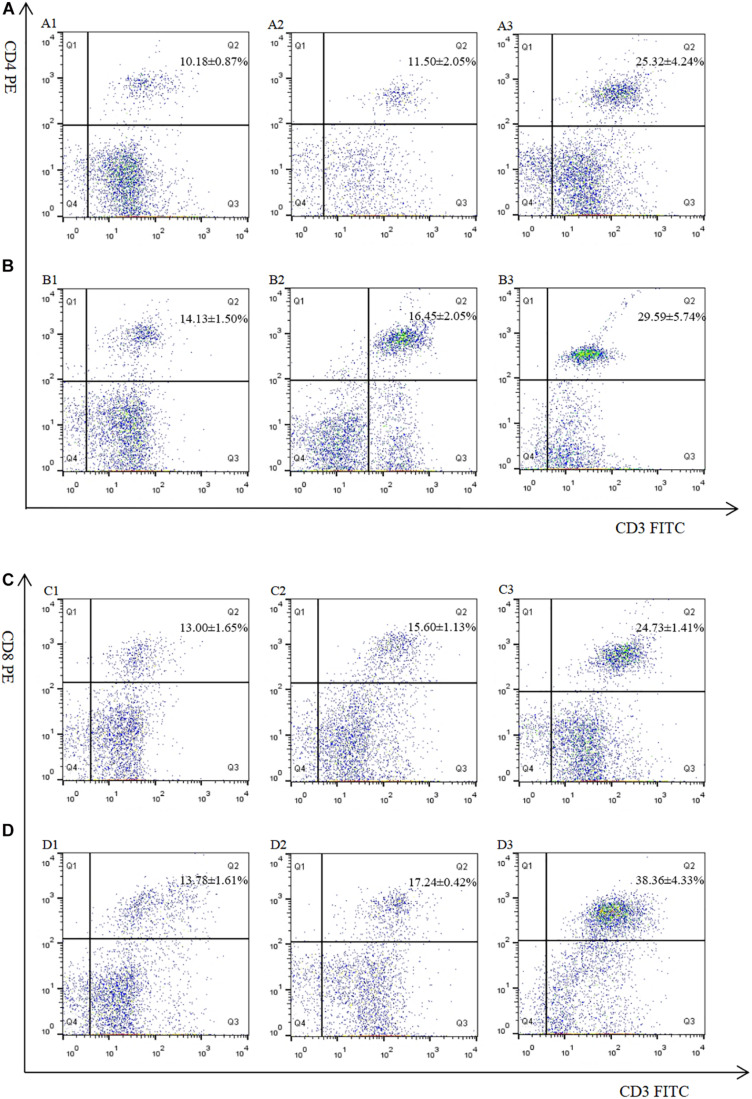
Percentage of T cell subpopulation in the spleen of pVAX1-EmROM3 immunized chickens. *N* = 5, the data = mean ± standard deviation. **(A)** Detection of CD4^+^/CD3^+^ T lymphocyte in immunized chicken spleen at 21 days old (7 days after the first immunization). **(B)** Detection of CD4^+^/CD3^+^ T lymphocyte in immunized chicken spleen at 28 days old (7 days after the booster immunization). **(C)** Detection of CD8^+^/CD3^+^ T lymphocyte in immunized chicken spleen at 21 days old (7 days after the first immunization). **(D)** Detection of CD8^+^/CD3^+^ T lymphocyte in immunized chicken spleen at 28 days old (7 days after the booster immunization). 1: PBS (negative control). 2: Detection of T lymphocytes with immunized plasmid pVAX1. 3: Detection of T lymphocytes with immunized pVAX1-EmROM3.

**FIGURE 5:**
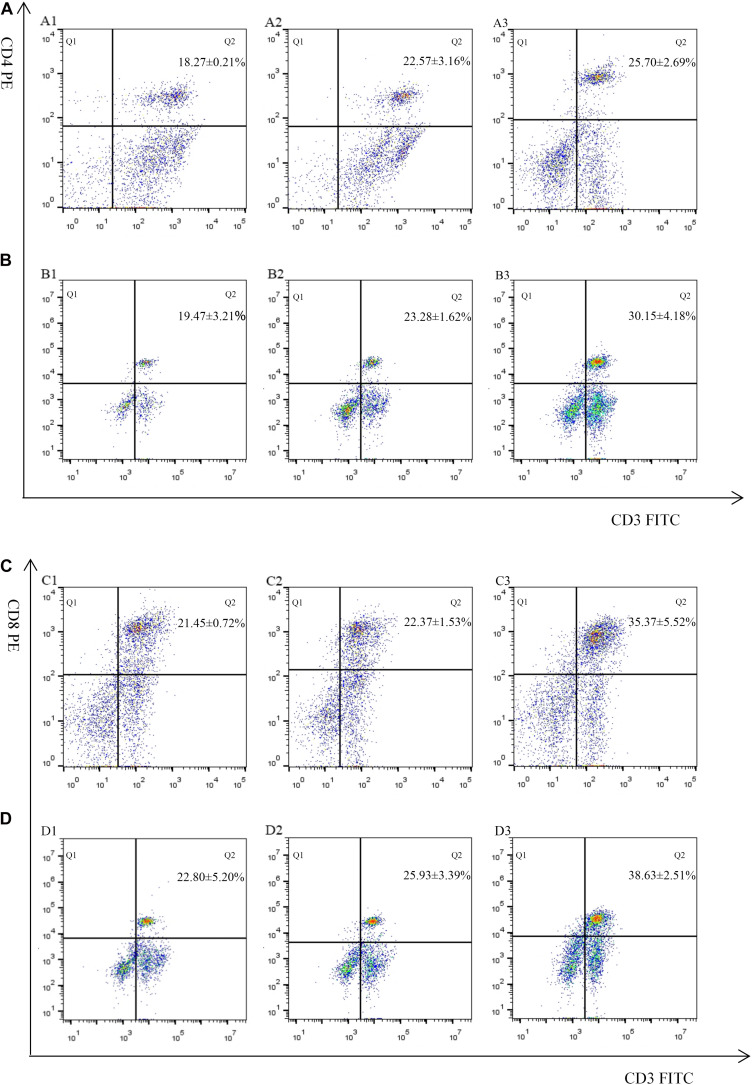
Percentage of T cell subpopulation in the spleen of recombinant protein pET-32a-EmROM3 immunized chickens. *N* = 5, the data = mean ± standard deviation. **(A)** Detection of CD4^+^/CD3^+^ T lymphocyte in immunized chicken spleen at 21 days old (7 days after the first immunization). **(B)** Detection of CD4^+^/CD3^+^ T lymphocyte in immunized chicken spleen at 28 days old (7 days after the booster immunization). **(C)** Detection of CD8^+^/CD3^+^ T lymphocyte in immunized chicken spleen at 21 days old (7 days after the first immunization). **(D)** Detection of CD8^+^/CD3^+^ T lymphocyte in immunized chicken spleen at 28 days old (7 days after the booster immunization). 1: PBS (negative control). 2: Detection of T lymphocytes with immunized tag protein pET-32a. 3: Detection of T lymphocytes with immunized recombinant protein pET-32a-EmROM3.

The proportion of CD4^+^ and CD8^+^ T cell subsets of experimental groups was measured 7 days after the first and second immunization by flow cytometry separately. As shown in Figures 4, 5 and Table 3, immunization with the recombinant protein of pET-32a-EmROM3 or recombinant plasmid of pVAX1-EmROM3 significantly promoted the proportion of CD4^+^ and CD8^+^ T cells compared with the controls (*p* < 0.05). The proportion of CD4^+^ and CD8^+^ T cells between the PBS control and tag protein control as well as empty plasmid control did not show a significant difference (*p* > 0.05).

**TABLE 3:** The proportion of T cell subpopulation in the spleen of immunized chickens.

Marker	Group	1st immunization	2nd immunization
CD4^+^/CD3^+^	PBS	10.18 ± 0.87^*a*^	14.13 ± 1.50^*a*^
	pVAX1 empty plasmid	11.50 ± 2.05^*a*^	16.45 ± 2.05^*a*^
	pVAX1-EmROM3 recombinant plasmid	25.32 ± 4.24^*c*^	29.59 ± 5.74^*c*^
CD8^+^/CD3^+^	PBS	13.00 ± 1.65^*a*^	13.78 ± 1.61^*a*^
	pVAX1 empty plasmid	15.60 ± 1.13^*a*^	17.24 ± 0.42^*a*^
	pVAX1-EmROM3 recombinant plasmid	24.73 ± 1.41^*c*^	38.36 ± 4.33^*c*^
CD4^+^/CD3^+^	PBS	18.27 ± 0.21^*a*^	19.47 ± 3.21^*a*^
	pET-32a tag protein	22.57 ± 3.16^*b*^	23.28 ± 1.62^*a*^
	rEmROM3	25.70 ± 2.69^*c*^	30.15 ± 4.18^*c*^
CD8^+^/CD3^+^	PBS	21.45 ± 0.72^*a*^	22.80 ± 5.20^*a*^
	pET-32a tag protein	22.37 ± 1.53^*a*^	25.93 ± 3.39^*a*^
	rEmROM3	35.37 ± 5.52^*c*^	38.63 ± 2.51^*c*^

**N* = 5, the data = mean ± standard deviation. Significant difference (*p* < 0.05) between data was annotated with different letters. No significant difference (*p* > 0.05) between data was annotated with the same letter.*

**FIGURE 6:**
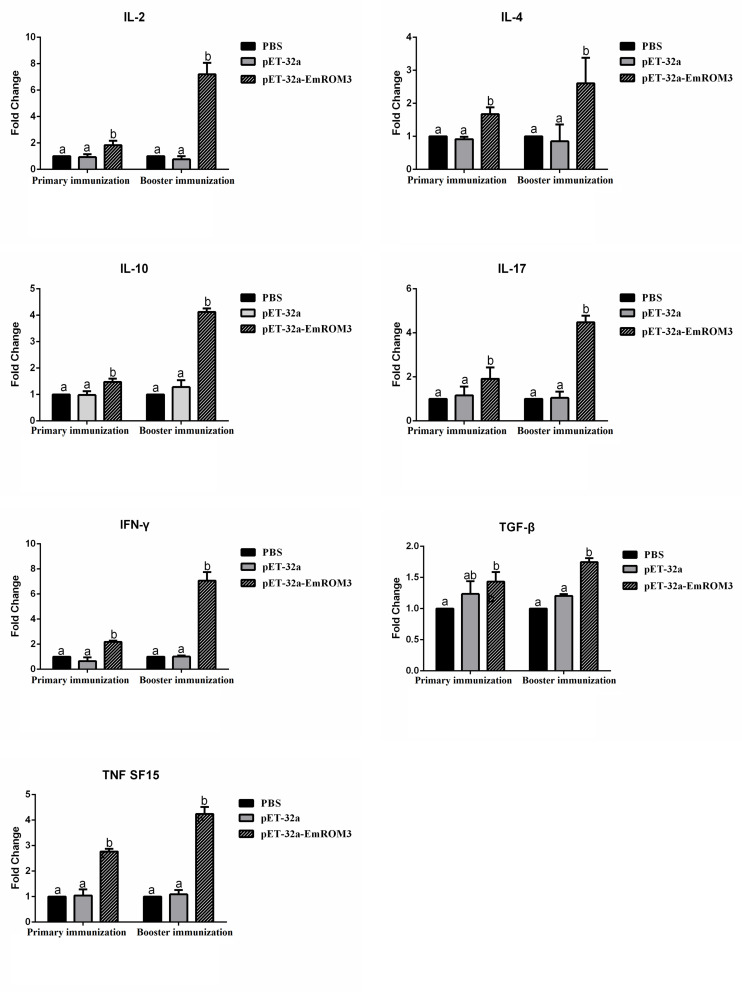
Transcript level of cytokine gene in sera from chickens immunized with recombinant protein pET-32a EmROM3. *N* = 5, the error bars = standard deviation. Significant difference (*P* < 0.05) between groups is marked with different letters. No significant difference (*P* > 0.05) between groups is marked with the same letter.

**FIGURE 7:**
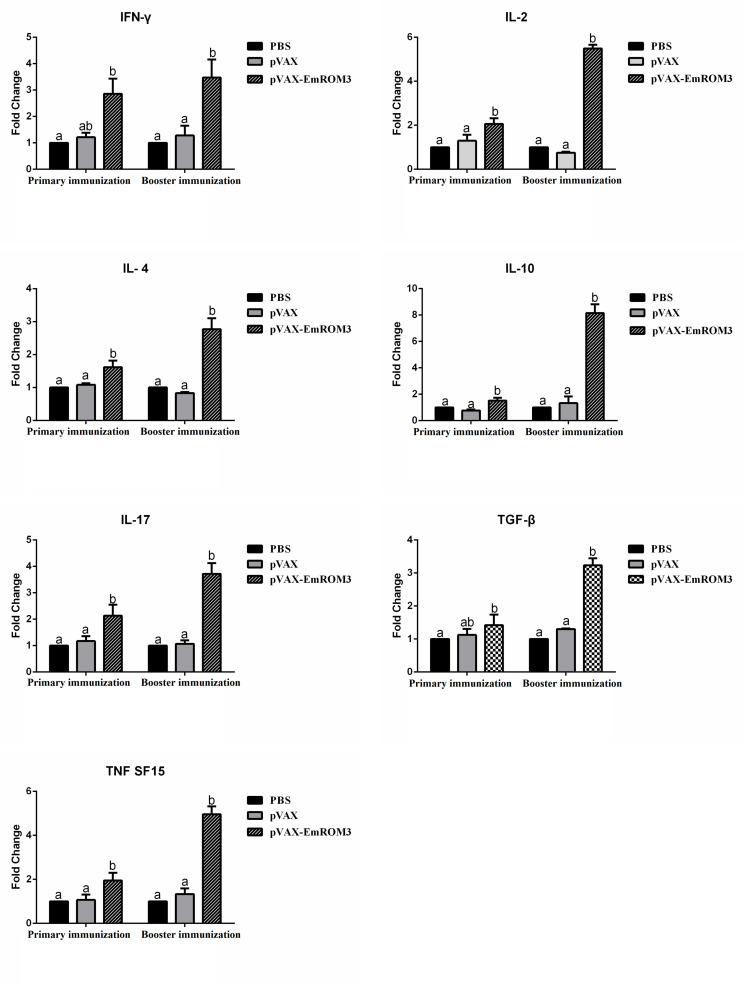
Transcript level of cytokine gene in sera from chickens immunized with plasmid pVAX-EmROM3. *N* = 5, the error bars = standard deviation. Significant difference (*P* < 0.05) between groups is marked with different letters. No significant difference (*P* > 0.05) between groups is marked with the same letter.

The transcription of cytokines IFN-γ, IL-2, IL-4, IL-17, TNFSF15, IL-10, and TGF-β4 from the immunized chicken were measured 7 days after the first and second immunization by qPCR separately. The result of qPCR (Figures 6, 7) indicated that the transcription level of the cytokines in the immunized groups was significantly higher compared to the PBS control as well as pET-32a tag protein or pVAX1 control (*p* < 0.05) 7 days after the first and second immunization. While the transcription level of the cytokines between the PBS control, pET-32a tag protein control as well as pVAX1 vector control did not show a significant difference (*p* > 0.05).

### Protective Efficacy of Recombinant ROM3 Vaccines Against *Eimeria maxima*

Protective efficacies of rEmROM3 and recombinant plasmid of pVAX1-EmROM3 were measured based on weight gain, oocyst production, lesion score, and ACI. Weight gain in the unchallenged control group was significantly higher than that in the challenged control group and tag protein control or plasmid control group (*p* < 0.05) (Table 2). Whereas the groups immunized with rEmROM3 or pVAX1-EmROM3 plasmid exhibited a significant restoration in body weight gain compared with the challenged control group and empty plasmid or protein control group (*p* < 0.05). A comparison between the experimental groups indicated that the body weight gain of rEmROM3, to a certain extent, is higher than that of the pVAX1-EmROM3 recombinant plasmid.

In this study, we used a numerical scale from 0 to 4 (normal to severe) to measure the enteric lesion score. When compared to the challenged control as well as tag protein and plasmid control, experimental chickens showed significant alleviations in enteric lesions (*p* < 0.05) (Table 2). The differences between chickens immunized with tag protein or plasmid were not statistically significant as compared with the challenge group (*p* > 0.05), which indicated that neither pET-32a tag protein nor pVAX1 plasmid plays a role in immune protection. The result of OPG shows a similarity with the enteric lesion score. Immunization with rEmROM3 or pVAX1-EmROM3 led to lower oocyst shedding and higher oocyst decrease ratio compared with pET-32a or pVAX1 controls, respectively (Table 2).

The data of ACI (Table 2) revealed a non-significance relationship between the challenged control, while the control group immunized with pET-32a tag protein and pVAX1 vector indicated that neither of them works as a protection against *E. maxima*. Two experimental groups, vaccinated with rEmROM3 or pVAX1-EmROM3, respectively, resulted in ACIs of 166.77 and 161.44, which were significantly higher compared with the challenged control, pET-32a tag protein, and pVAX1 plasmid control.

## Discussion

Coccidiosis remains a great threat to commercial poultry production as it brings tremendous loss worldwide (Blake and Tomley, 2014). Currently, the major control methods of coccidial infection are feeding anticoccidial drugs and vaccination with live vaccines. Although the use of drugs and live vaccines has greatly reduced, the mortality caused by coccidiosis and use of vaccines is increasingly restricted due to drug resistance, drug residue, and the high cost of producing live anticoccidial vaccines, etc (Shirley et al., 2007; Vanessa et al., 2014). Novel anti-coccidiosis vaccines such as subunit and DNA vaccines are considered as a promising control strategy against the *Eimeria* species. Compared with traditional vaccines, these novel vaccines are safer, cheaper, more simple to prepare, and more convenient in terms of storage and transportation (Dhama et al., 2008; Vanessa et al., 2014).

Identifying feasible vaccine candidates has always been a crucial and difficult task in preparing subunit vaccine and DNA vaccines. The proteins involved in host-cell invasion have been proposed as promising vaccine candidates (Vermeulen, 1998). Microneme proteins (MICs), for example, function in the adhesion to the host cell during invasion by coccidian parasites and exhibit promising protective efficacy against infection by *Eimeria* species (Carruthers and Tomley, 2008; Cowper et al., 2012; Li et al., 2020). Lai et al. (2011) reported that MIC3 from *Eimeria tenella* (EtMIC3) contributed greatly to the cecal tissue tropism of *E. tenella* in early invasion (Lai et al., 2011). Furthermore, they demonstrated that the recombinant protein and DNA of EtMIC3 are effective vaccine candidates in a preliminary immunization trial. Sun et al. (2014) evaluated the protective efficacy of MIC2 of *Eimeria tenella* (EtMIC2) delivered in *Saccharomyces cerevisiae* and found it offered partial protection against challenge infection by *E. tenella* (Sun et al., 2014). Huang et al. (2018) demonstrated that MIC3 from *Eimeria mitis* (EmiMIC3) was a promising vaccine candidate in an immunization-challenge animal experiment. Since ROMs are involved in the shedding of the MICs adhesins, several ROMs were tested as antigen candidates and exhibited promising protective efficacy. Earlier, an *E. tenella* rhomboid gene belonging to the ROM5 class was ligated to the fowlpox virus vector pUTA2 in efforts to select therapeutic targets against homologous infection (Yang et al., 2008). Another DNA vaccine carrying the fragment of the rhomboid gene by *Mycobacterium bovis* BCG developed a novel vaccine vector for further improvement of immunization strategies (Wang et al., 2009). More recently, the immunoprotective effect of a nucleic acid vaccine containing *Cryptosporidium baileyi* rhomboid gene was investigated and the data showed a certain protective immunity against Cryptosporidium infection with a high dose (Yang et al., 2016). These works demonstrated the feasibility of rhomboid protease as a candidate vaccine antigen. In this study, we demonstrated that rhomboid-like protein 3 from *E. maxima* provided partial protection against infection by *E. maxima* in forms of recombinant protein or eukaryotic expression plasmid, suggesting that EmROM3 protein and DNA are effective vaccine candidates.

Host cell invasion by apicomplexan parasites requires host adhesive proteins (MICs) to recognize host intestinal cells (Lai et al., 2011; Cowper et al., 2012). Shedding of the adhesins MICs by proteolytic cleavage is a pivotal step to disengage the parasite from the host cell and allow successful invasion (Dogga and Soldati-Favre, 2016). Rhomboid proteases (ROMs) are thought to be responsible for the cleavage of MICs from the host cell and completely enter the host cell (Dowse et al., 2005; Shen et al., 2014). Adhesins-cleaving characteristics differ among different apicomplexan parasites. In *Toxoplasma gondii*, TgROM2 was reported to cleave chimeric proteins of TgMIC2 and TgMIC12 (Dowse et al., 2005). Brossier et al. (2005) reported that MIC adhesins were cleaved by TgROM5 in a cell-based assay. However, Shen et al. (2014) reported that ROM4, and not ROM5, plays an essential role in adhesin cleavage using ROM-defected *T. gondii* tachyzoites. In *Plasmodium*, PfROMs 1 and 4 were found to be able to cleave most of the reported adhesins proteins involved in host invasion by *Plasmodium* (Dowse and Soldati, 2005). In *E. tenella*, ROM3 was reported to be involved in the cleavage of EtMIC4 (microneme protein 4) (Zheng et al., 2014). We have previously found that *Eimeria maxima* ROM3 (EmROM3) had a potential role in EmMICs cleavage (unpublished data). In this study, we found that vaccination with EmROM3 induced high titer of specific IgG and conferred partial protection against challenge infection by *E. maxima*. We speculate that the specific antibody induced by EmROM3 might inhibit the cleavage of EmMICs by EmROM3, thus interfering with the invasion of the parasite. Our results indirectly demonstrate the essential role of ROM3 in host invasion by *E. maxima*. However, more detailed experiments are needed to verify the exact situation.

It has been well recognized that cellular immunity dominated in conferring protection against coccidiosis (Rose et al., 1992; Trout and Lillehoj, 1996; Constantinoiu et al., 2008). Both CD8^+^ and CD4^+^ T lymphocyte subsets are involved. CD8^+^ T lymphocytes act as effector cells or secrete cytokines to mediate anticoccidial immunity in chickens. CD4^+^ T lymphocytes are mainly responsible for the production of helper cytokines (Vanessa et al., 2014; Kim et al., 2019). Many experimental results revealed that the proportion of CD8^+^ and CD4^+^ T lymphocyte subsets significantly increased in *Eimeria* species-infected chickens (Bessay et al., 1996; Vermeulen, 1998). Ma et al. (2017) reported that the levels of CD4^+^ and CD8^+^ T lymphocytes increased in spleens of chickens immunized with *E. tenella* 3-1E protein (Bessay et al., 1996). In this study, we obtained a similar result. The proportion of CD8^+^/CD3^+^ and CD4^+^/CD3^+^ T cells from spleens of experimental chickens showed a significant increase, which indicated a stimulation to the host that the EmROM3 induced for the production of cell-mediated immunity against coccidiosis and induction of T cell proliferation and differentiation.

T lymphocytes mediate the antigen-specific protection against infection with *Eimeria* by producing numerous cytokines, such as Th1, Th2, Th17, Treg, and proinflammatory cytokines, etc (Lillehoj and Lillehoj, 2000; Lillehoj et al., 2004; Kim et al., 2019). Among all the cytokines mentioned above, Th1 cytokines are the most efficient against avian coccidiosis (Kim et al., 2019). In this study, the transcript level of hallmark cytokines of Th1 cytokines (IL-2 and IFN-γ) significantly increased at 7 days after the first and second immunization, indicating a robust Th1 response induced by EmROM3. Besides, the Th1 cytokines, other types of cytokines like Th2, Th17, and proinflammatory cytokines are also important against avian coccidiosis (Lee et al., 2010; Kim et al., 2019). IL-4, a hallmark of Th2 cytokines, is considered to function by inducing a host humoral immune response against avian coccidiosis (Fallon et al., 2002; Dalloul and Lillehoj, 2006). IL-17, a signature cytokine produced by Th17 cells, is involved in the production of cytokines particularly IL-6, CXCL8, and GM-CSF, and exerts a proinflammatory role in *Eimeria* infection (Harrington et al., 2005; Hwang et al., 2008; Bystrom et al., 2013; Min et al., 2013; Peters and Yosef, 2014). TNF is a primary proinflammatory cytokine that plays crucial roles in modulating the immune response during the acute-stage of infection by *Eimeria* species (Allen and Fetterer, 2002; Moraes et al., 2019). In the present study, IL-4, IL-17, and TNFSF15 significantly increased in ROM3-immunized chickens, which agrees with previous reports and indicates that they play important roles in protection against *Eimeria* infection. Although inflammatory response is helpful in resisting coccidiosis in the early stages of coccidial infection, an excessive inflammatory response might cause intestinal injury. Treg cytokines like IL-10 and TGF-β might alleviate intestinal injury by inhibiting the inflammatory response. Thus, the result of an inflammatory response during *Eimeri*a infection depends on the balanced interplay between Treg and proinflammatory cytokines (Sehrawat and Rouse, 2017; Kim et al., 2019). In addition, the upregulated TGF-β is helpful for the repair of intestinal injuries following infection (Dalloul and Lillehoj, 2006). This was perhaps what caused an increase in the transcript level of the Treg cytokines of IL-10 and TGF-β from the immunized chickens observed in this study.

The role that humoral immunity plays during *Eimeria* species invasion, however, is controversial, with a large body of evidence indicating a minor function (Lillehoj and Trout, 1996; Yun et al., 2000). Nevertheless, the antibody was considered to be of some relevance in immune protection against coccidiosis in several recent studies (Belli et al., 2004; Ding et al., 2004; Constantinoiu et al., 2008). A typical example is the first commercial anticoccidial subunit vaccine of CoxAbic^®^ (Netanya, Israel), which is delivered via maternal immunization. Offspring chicks could inherit transmission-blocking immunity against *E. maxima* infection by vaccinating the breeding hens with CoxAbic^®^ composed of the microgametocyte antigens of *E. maxima* (Sharman et al., 2010). In our study, the specific IgG antibody levels in the EmROM3-immunized chickens significantly increased. We speculate that the upregulated antibody induced by EmROM3 might be involved in protection against *E. maxima* challenge by interfering with the invasion of the parasite.

## Data Availability Statement

The datasets presented in this study can be found in online repositories. The names of the repository/repositories and accession number(s) can be found in the article/Supplementary Material.

## Ethics Statement

The animal study was reviewed and approved by the Committee on Experimental Animal Welfare and Ethics of Nanjing Agricultural University.

## Author Contributions

XS and XrL: conceptualization. DT: investigation and methodology. LX, XrL, XS, and RY: resources. DT and CC: software. XS: supervision. CC and LX: validation. JS and XqL: visualization. CC: manuscript writing of the original draft. XS, CC, and MS: manuscript writing, review, and editing. All authors contributed to the article and approved the submitted version.

## Conflict of Interest

The authors declare that the research was conducted in the absence of any commercial or financial relationships that could be construed as a potential conflict of interest.

## Footnotes

1 https://blast.ncbi.nlm.nih.gov/Blast.cgi
