# Supplementary figures and images for: Protective Efficacy of Rhomboid-Like Protein 3 as a Candidate Antigen Against Eimeria maxima in Chickens
Source: Front Microbiol. 2021 May 5;12:614229. doi: 10.3389/fmicb.2021.614229 (PMC8131851; doi:10.3389/fmicb.2021.614229)

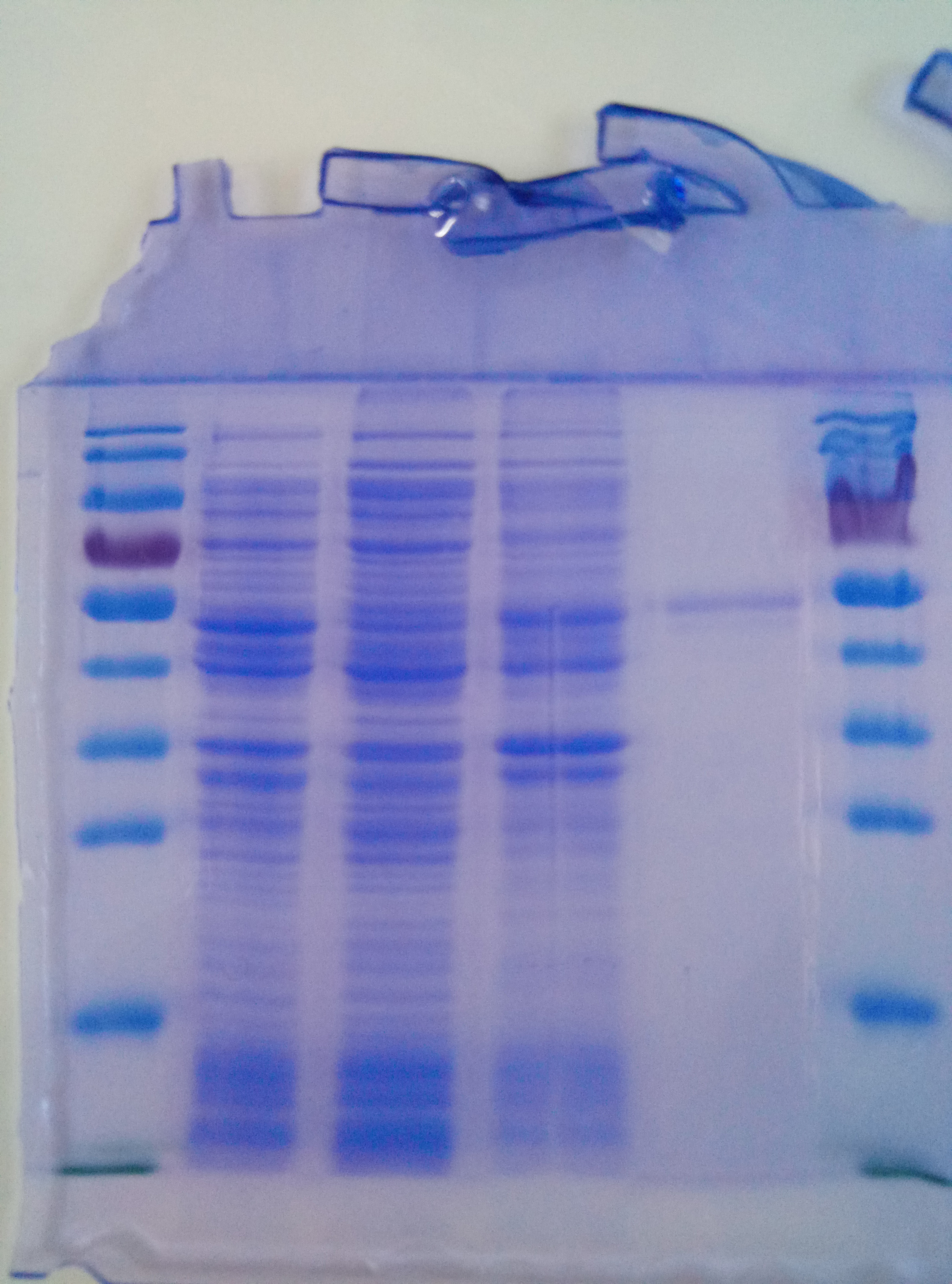

Supplement: Supplementary file 1 [file Image_1.JPEG]

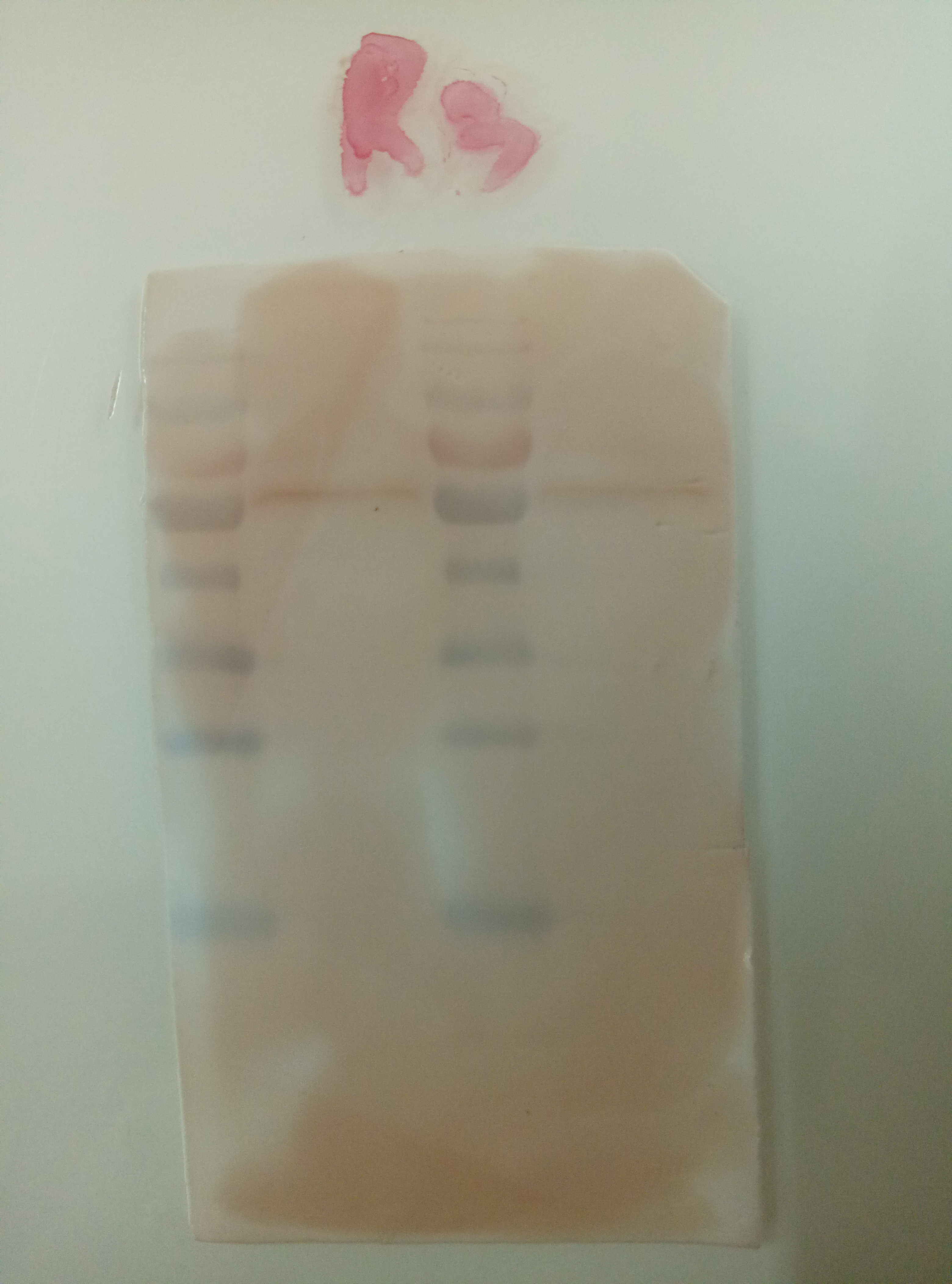

Supplement: Supplementary file 2 [file Image_2.JPEG]

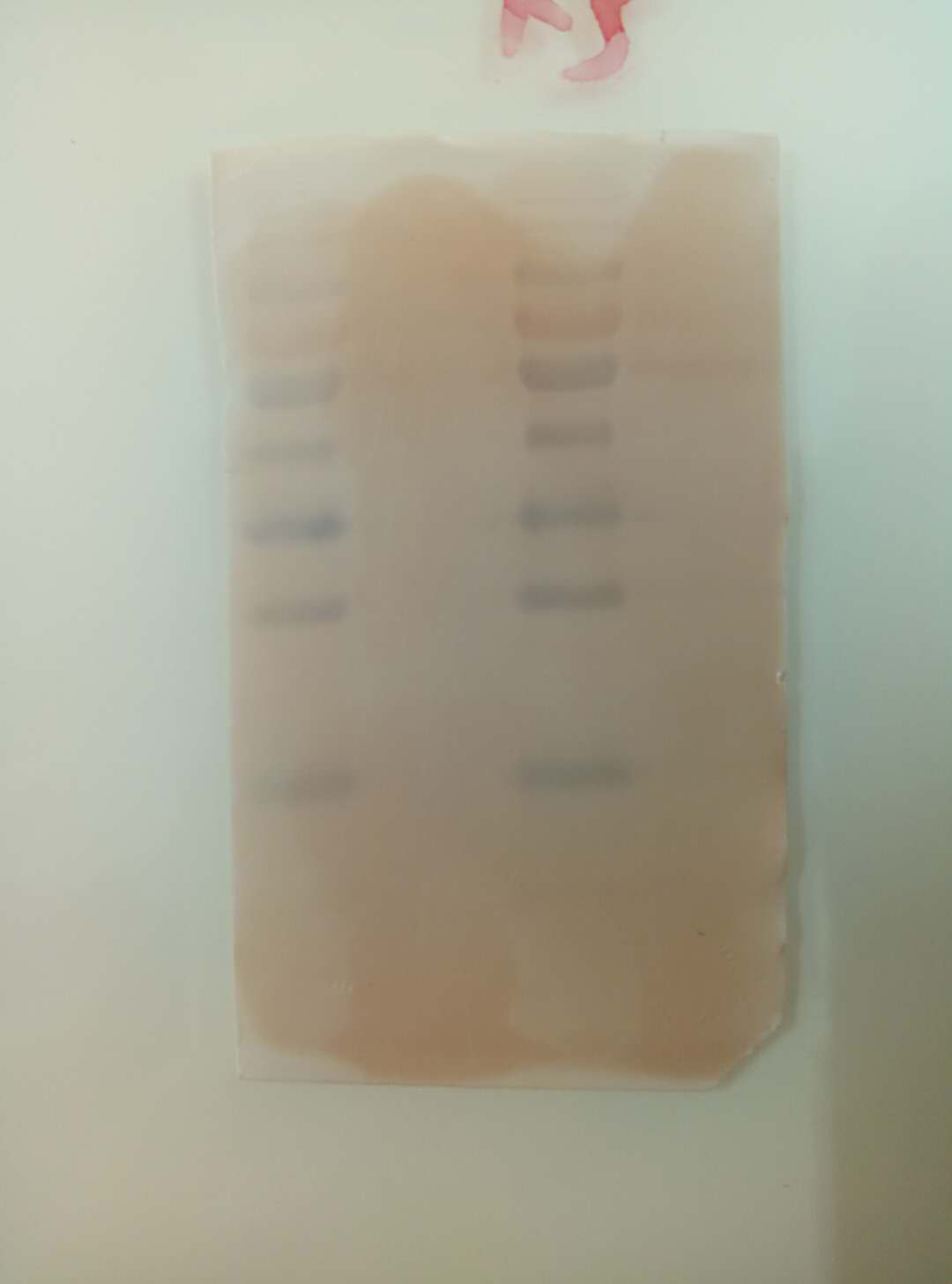

Supplement: Supplementary file 3 [file Image_3.JPEG]

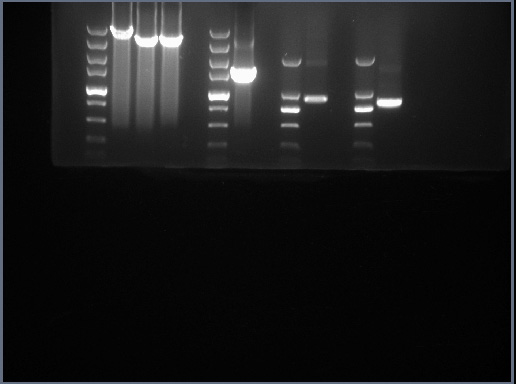

Supplement: Supplementary file 4 [file Image_4.JPEG]

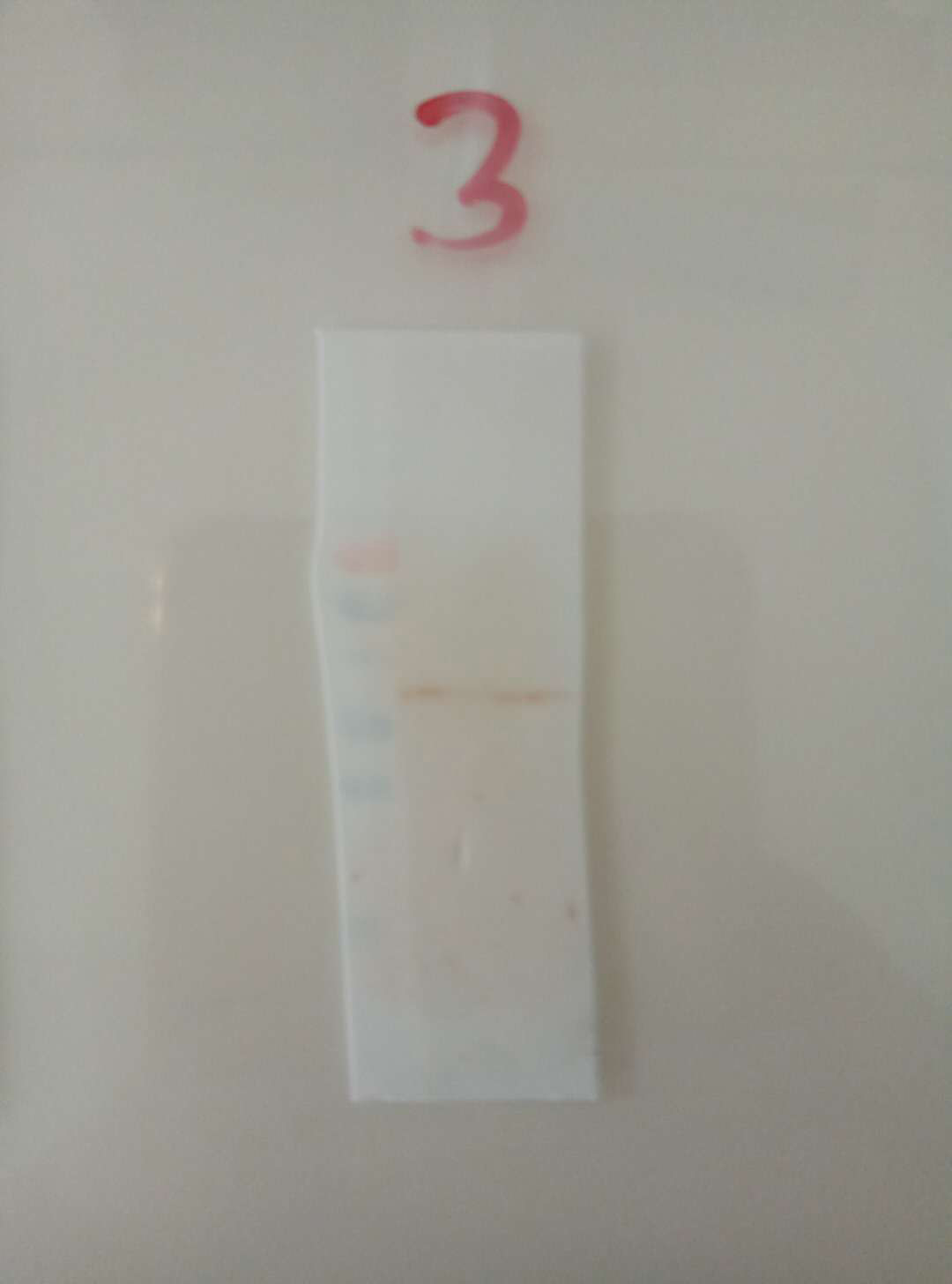

Supplement: Supplementary file 5 [file Image_5.JPEG]

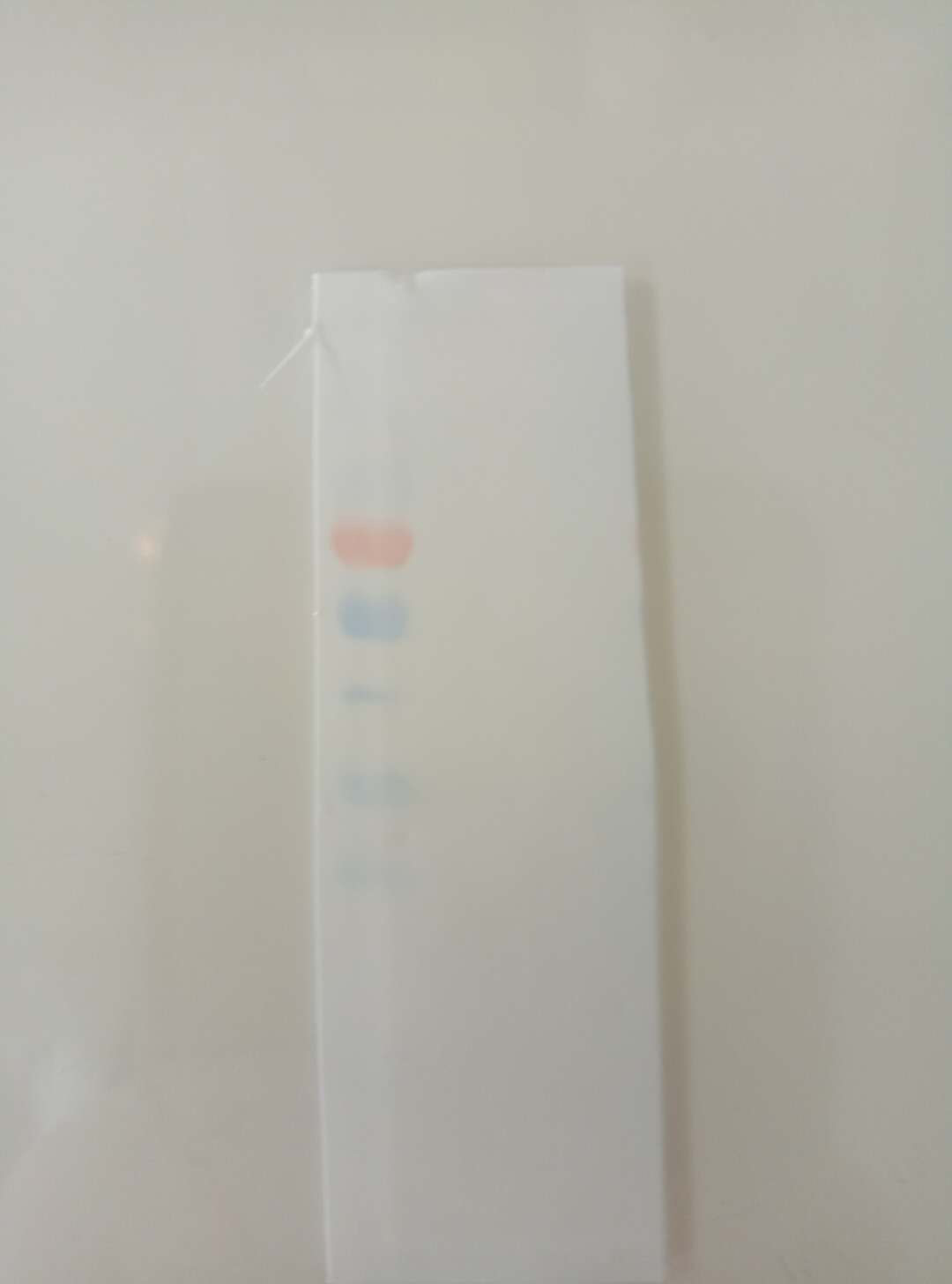

Supplement: Supplementary file 6 [file Image_6.JPEG]
